# Supplementary figures and images for: Spatial-temporal assessment of future population exposure to compound extreme precipitation-high temperature events across China
Source: PLoS One. 2024 Aug 14;19(8):e0307494. doi: 10.1371/journal.pone.0307494 (PMC11324101; doi:10.1371/journal.pone.0307494)

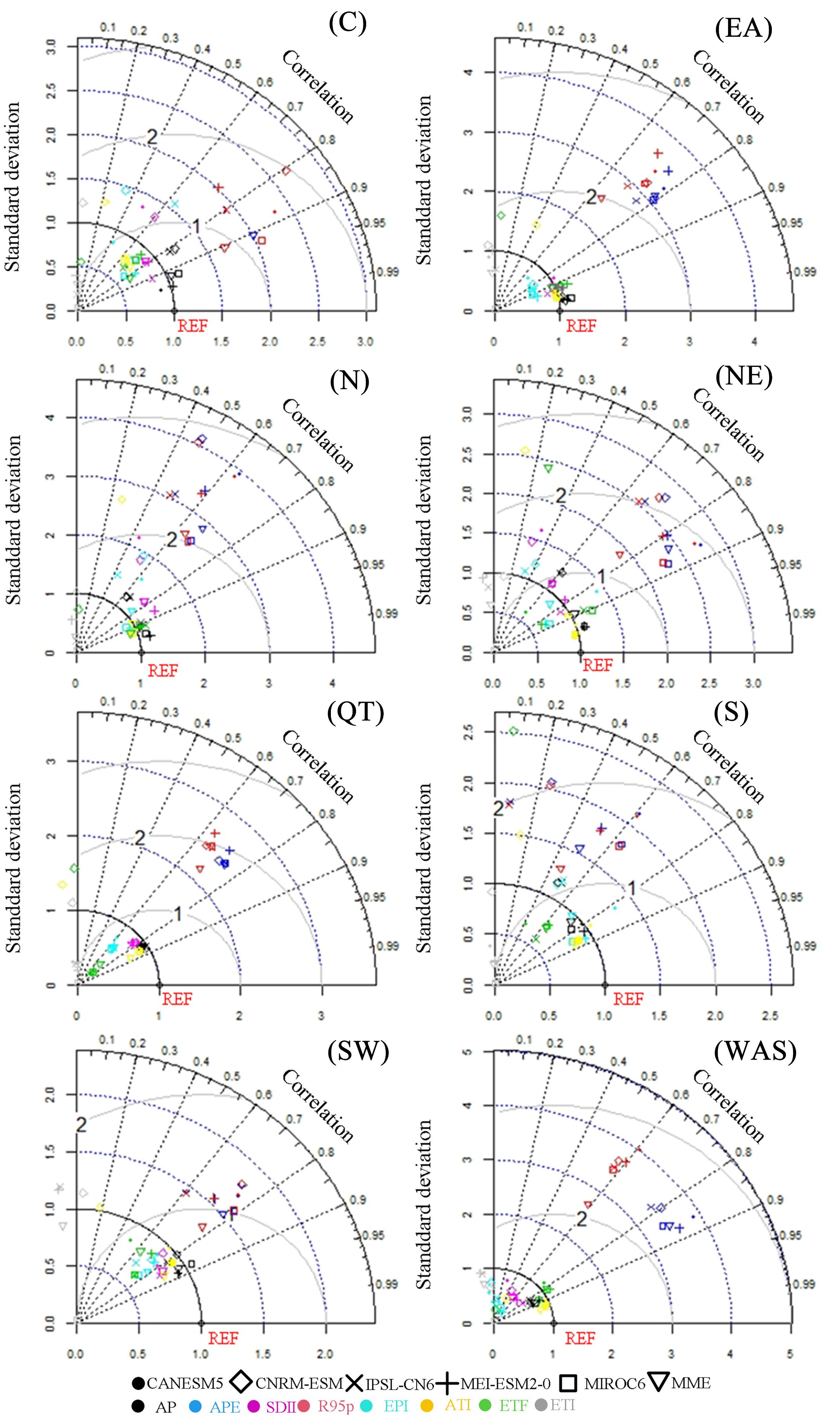

Supplement: S1 Fig — (TIF) [file pone.0307494.s001.tif]

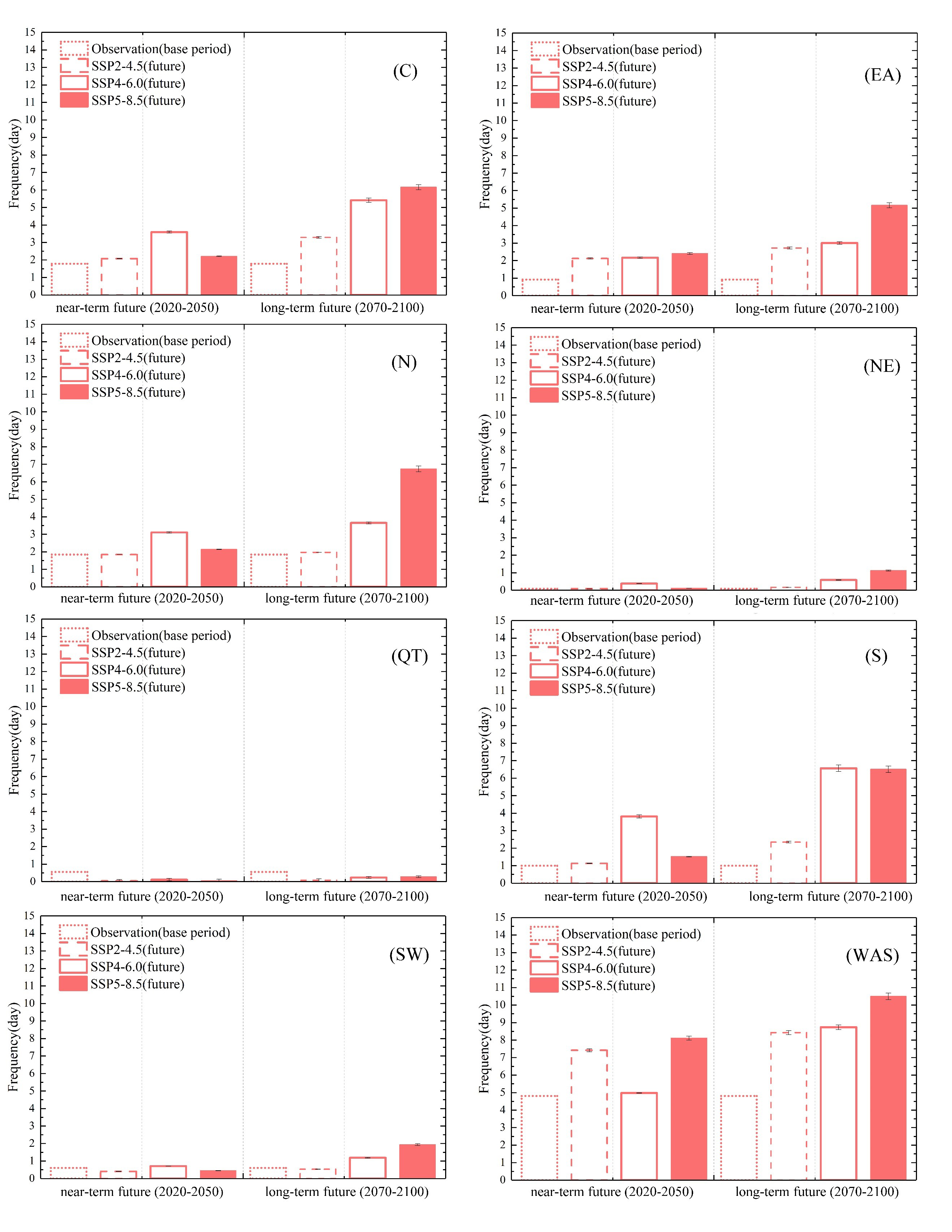

Supplement: S2 Fig — (TIF) [file pone.0307494.s002.tif]

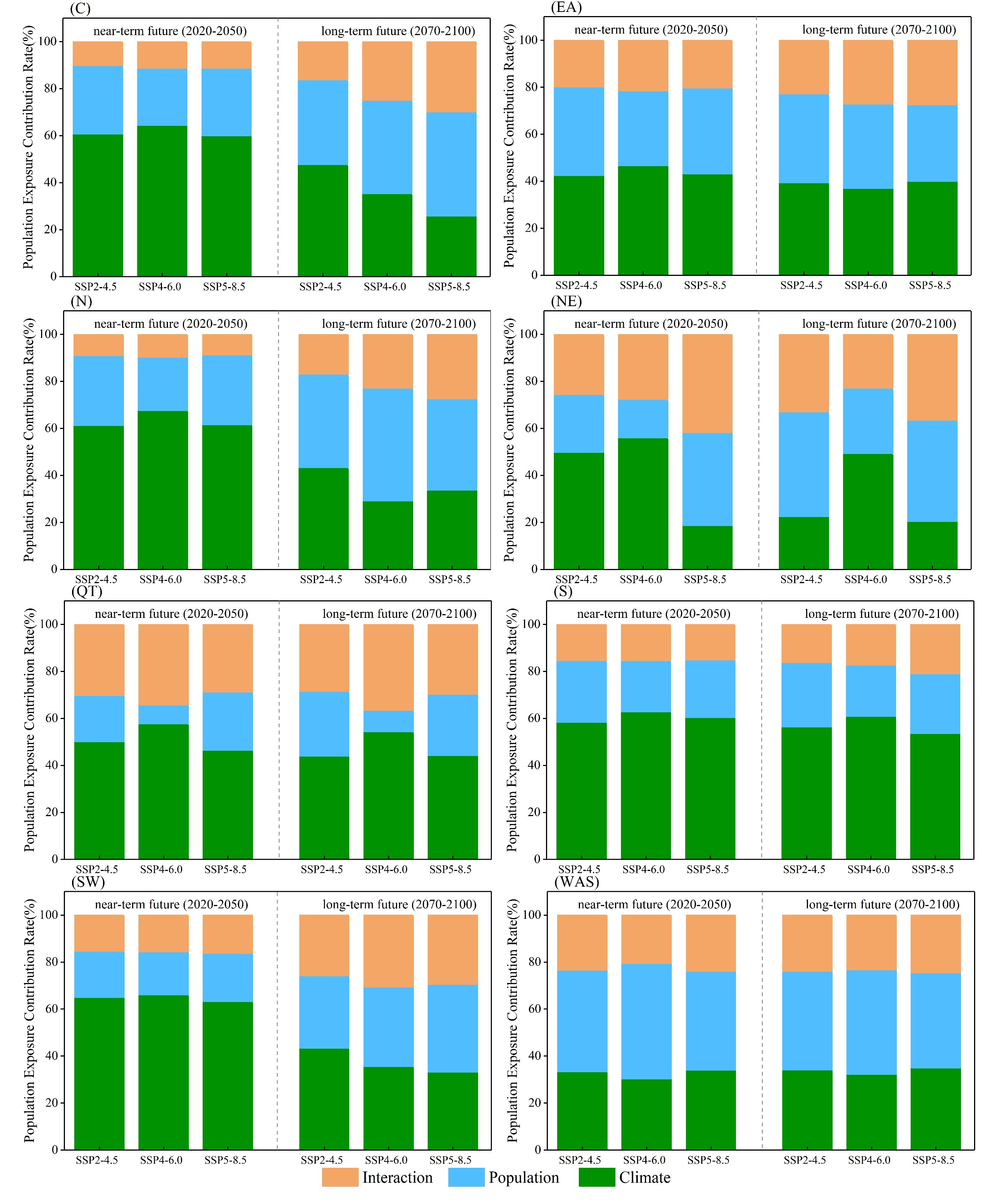

Supplement: S3 Fig — (TIF) [file pone.0307494.s003.tif]
