# Supplementary material for: Spatial-temporal assessment of future population exposure to compound extreme precipitation-high temperature events across China
Source: PLoS One. 2024 Aug 14;19(8):e0307494. doi: 10.1371/journal.pone.0307494 (PMC11324101; doi:10.1371/journal.pone.0307494)
Supplement: Attachment — Submitted filename: response letter.docx [file pone.0307494.s004.docx]

Response to the Manuscript entitled " Spatial-temporal assessment of future population exposure to compound extreme precipitation-high temperature events across China"

Dear editor:

This study, entitled "Spatial-temporal assessment of future population exposure to compound extreme precipitation-high temperature events across China", utilizes CMIP6 to predict spatiotemporal variations of compound extreme precipitation-high temperature events in China across different future scenarios. Despite CMIP6 effectively reproducing precipitation patterns, biases are observed. The frequency of compound events increases, particularly under high radiative forcing, with economically developed areas emerging as hotspots. Notably, the research underscores a significant shift in population exposure to compound events in the long-term future (2070-2100), emphasizing the growing influence of population factors over climate factors. This aligns with PLOS ONE's commitment to multidisciplinary research on impactful global issues.

This manuscript, authored by Ke Jin^1,2,3^, Yanjuan Wu^1,2,3^, Xiaolin Sun^1,2,3^, Yanwei Sun^1,2,3^, Chao Gao^1,2,3*^, has not been previously published and is not under consideration elsewhere. All listed authors have reviewed and approved the content. This submission is exclusive to your journal.

(1 Department of Geography and Spatial Information Techniques, Ningbo University, Ningbo, China, 2 Zhejiang Collaborative Innovation Center & Ningbo Universities Collaborative Innovation Center for Land and Marine Spatial Utilization and Governance Research, Ningbo, China, 3 Donghai Academy, Ningbo University, Ningbo, China ^*^gaoqinchao1@163.com)

We sincerely appreciate the editor's time and thoughtful consideration of our manuscript. Thank you for the invaluable insights and guidance provided.

5. Review Comments to the Author

Please use the space provided to explain your answers to the questions above. You may also include additional comments for the author, including concerns about dual publication, research ethics, or publication ethics. (Please upload your review as an attachment if it exceeds 20,000 characters)

Reviewer #1: Review comments on “Spatial-temporal assessment of future population exposure to compound extreme precipitation-high temperature events across China” by Jin et al. This manuscript used CMIP6 to predict the spatial-temporal variations of future population exposure to compound extreme events in China under three SSPs. In addition, this study showed the relative contribution of the factors contributing to changes in population exposure. The topic and the results are interesting. However, there are a few more details to be discussed in this manuscript. Therefore, I recommend a major revision. My specific comments are as follows:

(1)Page 2 The first sentence: You refer to IPCC AR6, the citation should more appropriately changed to 2021.
**Response**: Thanks for the heads up, this oversight has been fixed!

“In accordance with the Sixth Assessment Report (AR6) from the Intergovernmental Panel on Climate Change (IPCC), the average global surface temperature increased by 0.99°C during the initial two decades of the 21st century (2001–2020), as measured against the pre-industrial period (IPCC, 2021).”

(2)Page 5: Why were these five models chosen and how did the other models in CMIP6 perform?
**Response**: We have added a section of discussion on the reasons for choosing them in the introduction of the manuscript.

Using multi-model ensemble mitigated internal variability within models, has been proved can effectively reducing the uncertainty associated with individual climate models (Xu et al., 2022). However, studies have indicated that an excessive number of models can introduce additional uncertainty during the simulation process (Liang et al., 2020; Ji et al., 2024). The increasing temperature levels were found to amplify inter-model uncertainty, especially within specific regions, raising concerns about excessive variability in extreme events (Vrac et al., 2023; Liu et al., 2021). To address this issue, selecting some models with superior simulation performance to fit together emerged as a favorable solution (Yang et al., 2021).

“The five models were chosen for their robust global climate simulation capabilities, demonstrating accuracy in depicting historical temperature and precipitation trends, which proved effective in capturing regional climates, particularly in China and Asia (Séférian et al., 2019; Christian et al., 2021; Niu et al., 2023; Tian et al.,2021). Scholars have demonstrated the advantages of accurately assessing climate change in the study region by assembling these climate models and the certain advantages over this group have been shown (Su et al., 2021; Andrade et al., 2021).”

(3)Page 6: Would similar results be obtained using the other population data?

**Response**: We apologize for the confusion, this dataset integrates different climate scenarios, enriches the diversity of factors in population estimation, comprehensively considers the roles of multiple climate change scenarios, and social development patterns in population, and finally summarizes the population estimation under different socio-economic development patterns (Jiang et al., 2022).
(4)Page 8: Definition of extreme precipitation thresholds not rigorous enough. Based on your description, you selected only the precipitation for the day in the study period (1970 - 2000) for ranking (the sample size is too small). You can expand the sample size by choosing 15 days of the study period, which corresponds to 7 days on either side of the target date.
 **Response**: A three-day time window can be a good reflection of the continuity between extreme events; moreover, expanding the time window too long can lead to a small sample size, making it difficult to conduct quantitative studies. Thank you very much for your input, we will explore other time windows further in the future.

“This study focuses on extreme precipitation events, which are defined as instances significantly deviating from climatic averages, typically resulting in high impact despite their low probability of occurrence. To identify these events, a spatiotemporal composite method is employed, integrating extreme precipitation, temperature, and high temperature criteria. Individual extreme events are determined using percentile thresholds. Specifically, for each grid point within the defined spatiotemporal scope, daily maximum precipitation values are sorted, with the 95th percentile serving as the threshold for extreme precipitation events. This establishes threshold values for extreme precipitation across historical and future periods. High temperature is a critical component in identifying compound extreme events. A threshold is set, designating a day as potentially high-temperature when the maximum temperature exceeds 35°C. Compound extreme precipitation-high temperature events are identified when both conditions are met: the maximum temperature surpasses 35°C at a specific grid point, and there is precipitation exceeding the relative threshold within a 3-day window. To ensure accuracy, a time window concept is applied. Following the occurrence of high-temperature conditions, the subsequent 3 days are examined for precipitation exceeding the relative threshold. This window accommodates potential lag effects between high temperature and precipitation, balancing continuity while avoiding a small sample size for quantitative.”
(5)Page 10: Fig 2 Taylor diagram and the corresponding text description do not match, please re-check and confirm. For example: Page 10 Line 2-3, the SCC of precipitation indices between all models and observations range, what is represented on Fig 2 is not consistent with the range you stated. And “the MRI-ESM2-0 and the MME exhibit SCC exceeding 0.80” do not match either. Line 11-12, please check the range of RMSE, which is generally non-negative……
 **Response**: We modified the values and reconstructed the graphs for our analysis.

“The mean values for each grid, covering both observations and historical modeled data from the base period (1970-2000), were computed. Following this, we evaluated how well five CMIP6 models simulated mean and extreme precipitation and temperature across China. We also assessed the multi-model ensemble (MME) using the Taylor diagram (Fig 2) for analysis. For precipitation indices, the SCC between all models and observations range from 0.42 to 0.97. Notably, the MRI-ESM2-0 and the MME exhibit SCC exceeding 0.80. The RMSE of simulations compared to observations range from 0.34 to 2.98. The SDR consistently exceeds 1, indicating favorable simulation performance of climate models for both the climatology and extremes of precipitation events. Consistent with this study, some researchers have observed an overestimation of spatial variability in precipitation frequency. This occurred when evaluating the performance of CMIP6 models in simulating precipitation across China. This overestimation may be associated with a positive bias in light precipitation frequency, suggesting that climate models simulate precipitation too frequently with insufficient intensity (Stephens et al., 2010; Wu et al., 2016).

For temperature indices, the SCC between all models and observations range from 0 to 0.98. Notably, the MRI-ESM2-0 and the MME exhibit SCC surpassing 0.86. The RMSE of simulations compared to observations ranges from 0 to 1.12. The SDR consistently exceeds 1, indicating satisfactory simulation performance of climate models for both the climatology and extremes of high-temperature events. Moreover, to conduct a more comprehensive assessment of CMIP6's simulation performance concerning precipitation and temperature, our analysis encompasses specific sub-regions (see Fig S1). There are notable variations in performance among different models across these diverse sub-regions. Regarding precipitation indices, the Multi-Model Ensemble (MME) exhibits exceptional performance in sub-regions like EA, NE, QT, S, and SW, whereas the MRI-ESM2-0 excels in C and N. Conversely, for temperature indices, except for IPSL-CM6A-LR, which excels in SW, the MME consistently outperforms in all other sub-regions. In summary, the MME consistently showcases impressive simulation capabilities throughout China, validating its ability to accurately replicate precipitation and temperature patterns at both regional and subregional scales.”

Fig 2 Taylor diagrams of precipitation and high-temperature index simulations by 5 climate models and the Multi-Model Ensemble (MME) for China from 1970 to 2000.

(6)Page 12 Fig 3: Please check why the observation values of the left and right sides of the figure are not consistent. In addition, the dashed lines on the left and right sides of the figure, which represent the SSP2-4.5 scenarios, are not of the same width. The same problem occurs in Fig 6.
 **Response**: Thanks for your comments, have made the changes.

Fig 3 The frequency of future compound extreme events in China for two periods (2020-2050 and 2070-2100) under historical conditions and three SSPs (SSP2-4.5, SSP4-6.0, SSP5-8.5)

Fig 6 The population exposed to compound events (unit: million person) of China in baseline period (1970-2000) and two future periods (2020-2050, 2070-2100) under 3 SSPs (SSP2-4.5, SSP4-6.0, SSP5-8.5).

(7)Page 15 Fig 4: Please check the text label on the left side of row 2, I guess you want to express "Change (2020-2050)". The same problem occurs in Fig 7.

**Response**: Thanks for your comments, have made the changes.

Fig 4 Compared to period 1970-2000, future frequencies (rows 1 and 3) and changes (rows 2 and 4) in compound events in China for two future periods (2020-2050 and 2070-2100) under three SSPs (unit: d/y)

Fig 7 Spatial distribution (the first and third rows) and changes (the second and fourth rows) of population exposed to compound events (unit: million person) of China in two future periods (2020-2050, 2070-2100) under 3 SSPs (SSP2-4.5, SSP4-6.0, SSP5-8.5) compared to baseline period (1970-2000).

Reviewer #2: The study titled “Spatial-temporal assessment of future population exposure to compound extreme precipitation-high temperature events across China” uses the outputs of multiple CMIP6 GCMs to estimate population exposure to compound temperature and precipitation extremes under three SSPs. The manuscript is well-written and presents interesting results, which would be of great interest to the readers. However, the following are some of my comments on the improvement of the manuscript, which I believe would improve its quality.
Comments:
Introduction:
• IPCC AR6 citation: IPCC 2012 >> IPCC 2022.

**Response**: Thank you for your offer, we've made changes.

“In accordance with the Sixth Assessment Report (AR6) from the Intergovernmental Panel on Climate Change (IPCC), the average global surface temperature increased by 0.99°C during the initial two decades of the 21st century (2001–2020), as measured against the pre-industrial period (IPCC, 2021).”

• The following papers are suggested for the authors to review for the introduction and discussion parts, as these papers also CMIP6 outputs for projections of independent and compound climate extremes in China and and neighboring regions. Compare and justify your results with the findings of these studies.
o https://doi.org/10.1016/j.scitotenv.2024.170133
o https://doi.org/10.1016/j.scitotenv.2023.162822
o https://doi.org/10.1016/j.atmosres.2023.106675
o https://doi.org/10.1088/1748-9326/ac9c70
**Response**: Thank you for your offer, we've made changes.

“Attention have been paid to this issue, some studies found that future drought-flood, water-flood, and drought-flood alternation events in China will increase significantly (Zhang et al., 2023). It was pointed out that the frequency of compound extreme drought and heatwave (CDHEs) in China in the future will be much higher than that of other compound events (Yu et al., 2023). In addition, not only extreme compound events but also mild weather events have received attention. In a warmer future, the occurrence of mild weather in southeastern China will decrease significantly compared to current levels. At the seasonal scale, the decrease in mild weather in summer outweighs the increase in spring and autumn (Zhang et al., 2022).”

• No keywords???

**Response**: We apologize for the confusion, there is no keyword requirement for this journal, the keywords originally set were CMIP6, composite climate extremes, climate change, China, etc.

• The section “Method” should be “Data and Method” and then put the other sub-headings under this main heading.

**Response**: We've modified this section
Data:
• I am wondering why the authors chose only five CMIP6 GCMs while we have dozens of GCMs. Also, they didn’t use the newly released high-resolution, statistically downscaled, and bias-corrected outputs of the NEX-GDDP-CMIP6 models, which are highly recommended for climate change impact assessment at a regional scale.

**Response**: We have added a section of discussion on the reasons for choosing them in the introduction of the manuscript.

Using multi-model ensemble mitigated internal variability within models, has been proved can effectively reducing the uncertainty associated with individual climate models. However, studies have indicated that an excessive number of models can introduce additional uncertainty during the simulation process (Liang et al., 2020; Ji et al., 2024). The increasing temperature levels were found to amplify inter-model uncertainty, especially within specific regions, raising concerns about excessive variability in extreme events. To address this issue, selecting some models with superior simulation performance to fit together emerged as a favorable solution.

The latest climate models and climate modifications are very valid and interesting, but due to technical constraints and time constraints it is difficult to process them at the moment, we will use these data in future studies and will keep you informed.

“The five models were chosen for their robust global climate simulation capabilities, demonstrating accuracy in depicting historical temperature and precipitation trends, which proved effective in capturing regional climates, particularly in China and Asia (Séférian et al., 2019; Christian et al., 2021; Niu et al., 2023; Tian et al.,2021). Scholars have demonstrated the advantages of accurately assessing climate change in the study region by assembling these climate models and the certain advantages over this group have been shown (Su et al., 2021; Andrade et al., 2021).”

• The authors used only three SSPs while overlooking SSP3, which is the most important one, particularly for estimating population exposure, as this SSP has been embedded with population effects. I would suggest to include SSP3 in the revision.
Method:

**Response**: Thank you for your comments, but due to data limitations and time constraints, only three pathways, SSP2-4.5, SSP4-6.0, and SSP5-8.5, are available in the current study, and the addition of the SSP3 pathway will be added in a future study.

• Table 2 is also named as Table 1. Please correct it.

**Response**: Table 1 has been changed to Table 2
• Why do the authors evaluate the GCMs' performance by simulating daily independent extreme temperature and precipitation, but not their compound extremes? Any logic or science behind it?

**Response**: Due to the many uncertainties associated with composite extreme events and the small sample size due to composite events, it is difficult to meet the statistical requirements. Therefore, only conventional metrics can be selected for GCM performance evaluation
• The definition of the compound extreme temperature and precipitation is not clear. Please rewrite it with more clear wording.

**Response**: We have made the following changes.

“This study focuses on extreme precipitation events, which are defined as instances significantly deviating from climatic averages, typically resulting in high impact despite their low probability of occurrence. To identify these events, a spatiotemporal composite method is employed, integrating extreme precipitation, temperature, and high temperature criteria. Individual extreme events are determined using percentile thresholds. Specifically, for each grid point within the defined spatiotemporal scope, daily maximum precipitation values are sorted, with the 95th percentile serving as the threshold for extreme precipitation events. This establishes threshold values for extreme precipitation across historical and future periods. High temperature is a critical component in identifying compound extreme events. A threshold is set, designating a day as potentially high-temperature when the maximum temperature exceeds 35°C. Compound extreme precipitation-high temperature events are identified when both conditions are met: the maximum temperature surpasses 35°C at a specific grid point, and there is precipitation exceeding the relative threshold within a 3-day window. To ensure accuracy, a time window concept is applied. Following the occurrence of high-temperature conditions, the subsequent 3 days are examined for precipitation exceeding the relative threshold. This window accommodates potential lag effects between high temperature and precipitation, balancing continuity while avoiding a small sample size for quantitative.”
Results:
• In bar graph figures, the bars can be shown in different colors, instead of blank bars with dashed and line bars.
• Figures 5 & 7, please put the unit either with the color bars or in the caption.

**Response**: We have made the following changes.

Fig 3 The frequency of future compound extreme events in China for two periods (2020-2050 and 2070-2100) under historical conditions and three SSPs (SSP2-4.5, SSP4-6.0, SSP5-8.5)

Fig 6 The population exposed to compound events (unit: million person) of China in baseline period (1970-2000) and two future periods (2020-2050, 2070-2100) under 3 SSPs (SSP2-4.5, SSP4-6.0, SSP5-8.5).

(7)Page 15 Fig 4: Please check the text label on the left side of row 2, I guess you want to express "Change (2020-2050)". The same problem occurs in Fig 7.

Fig 4 Compared to period 1970-2000, future frequencies (rows 1 and 3) and changes (rows 2 and 4) in compound events in China for two future periods (2020-2050 and 2070-2100) under three SSPs (unit: d/y)

Fig 7 Spatial distribution (the first and third rows) and changes (the second and fourth rows) of population exposed to compound events (unit: million person) of China in two future periods (2020-2050, 2070-2100) under 3 SSPs (SSP2-4.5, SSP4-6.0, SSP5-8.5) compared to baseline period (1970-2000).

• In limitations of the study, there should be some points on socioeconomic and demographic factors. See the following paper for reference.
o https://doi.org/10.1016/j.cliser.2022.100317
Discussion:
• The “Discussion” section can be extended a bit more, focusing on discussing/justifying the current results in light of the available literature.
• The following articles can be of good use for the authors to justify their results.
o https://doi.org/10.1016/j.wace.2023.100570
o https://doi.org/10.1029/2021EF002511
o https://doi.org/10.1029/2022EF003109
o <https://doi.org/10.1016/j.atmosres.2022.106554>

**Response**: Thanks to your comments, we have made the following changes

“Acknowledging limitations, our study highlights discrepancies between CMIP6 model simulations and actual data, affecting the accuracy of population risk predictions. Future research should leverage higher-resolution regional climate models and diverse population datasets to enhance robustness. Additionally, exploring the impacts of hydrological and land-use changes on extreme events remains a crucial avenue for further investigation. Rigorous validation of our results is imperative for a comprehensive understanding of the complex interactions influencing population exposure to extreme events. In selecting the sample population, it was not subdivided, and in fact some studies have shown that the impact of extreme events will be more significant for people who are older or younger (Shen et al., 2022). In addition, compound extreme events are not only harmful to the population, but also extremely costly to the socio-economy, which will be introduced in future studies.”

References
• The references list is incomplete and wrong. Some of the references are not cited correctly, i.e., ipcc >> IPCC
• Some are missing, i.e., (Shen et al., 2022)
• Likewise, many issues in the references.

**Response**: Thanks to your comments, we have made the following changes

Zhang Y, You Q, Ullah S, et al. Substantial increase in abrupt shifts between drought and flood events in China based on observations and model simulations[J]. Science of the Total Environment, 2023, 876: 162822.

Yu Y, You Q, Zuo Z, et al. Compound climate extremes in China: Trends, causes, and projections[J]. Atmospheric Research, 2023, 286: 106675.

Zhang J, You Q, Ren G, et al. Projected changes in mild weather frequency over China under a warmer climate[J]. Environmental Research Letters, 2022, 17(11): 114042.

Shen L, Wen J, Zhang Y, et al. Changes in population exposure to extreme precipitation in the Yangtze River Delta, China[J]. Climate Services, 2022, 27: 100317.

Your sincerely

Ke Jin (Ningbo University)

jinke505320@163.com
